# Seeing others suffer and enjoying it? The Model of Individual and Social Appraisals of Misfortunes of others in media reception

**DOI:** 10.3389/fpsyg.2024.1437721

**Published:** 2024-08-29

**Authors:** Lilian Suter, Katrin Döveling

**Affiliations:** ^1^School of Applied Psychology, ZHAW Zurich University of Applied Sciences, Zurich, Switzerland; ^2^Department of Communication and Media Research (IKMZ), University of Zurich, Zurich, Switzerland; ^3^Department of Social Sciences, University of Applied Sciences Darmstadt, Darmstadt, Germany

**Keywords:** emotion, schadenfreude, sympathy, Affective Disposition Theory, appraisal, media psychology

## Abstract

Suffering and misfortunes of other people are often portrayed in the media. Recipients react to these portrayals with different emotions. This article elucidates and clarifies schadenfreude (pleasure at the misfortune of others) and sympathy (feeling concern or sorrow over another person’s distress) in media experiences. A thorough literature review provides in-depth insights into the formation of affective dispositions and schadenfreude from various psychological perspectives. This conceptual analysis leads to the “Model of Individual and Social Appraisals of Misfortunes of Others” (MISAM) which first reveals the determining intrapersonal factors within the emotional experience of schadenfreude and sympathy. Second, it discloses the social component vital for understanding the construction and regulation of these emotions. The model combines individual and social appraisal processes and identifies the factors involved in the elicitation and regulation of schadenfreude and sympathy in the media reception of misfortunes. With the aim of integrating different perspectives, we incorporated Affective Disposition Theory and recent work from social psychology and used an appraisal framework. The MISAM opens the path for further investigation of schadenfreude and sympathy in media reception, beyond entertainment experiences.

## 1 Introduction

Laughing at others’ mishaps when watching reality TV, enjoying news about the downfall of a politician one does not agree with, or smiling at a social media feed that shows how one’s ex-boyfriend was dumped by his new girlfriend—all these situations describe people experiencing schadenfreude, “the pleasure at the misfortunes of others” (van Dijk and Ouwerkerk, 2014a, p. 6). This term combines joy (*freude*) with damage or harm (*schaden*) experienced by another person.

Schadenfreude is considered a discrete emotion and has been proposed as 1 of 16 enjoyable emotions by Ekman (2003). Thus, it is seen as “pleasurable to experience” (Graham et al., 2019, p. 207) and therefore potentially entertaining. At the same time, schadenfreude is characterized by a “disregard for others’ wellbeing” (Graham et al., 2019, p. 207) and labeled as belonging to the family of hazardous emotions. Reacting with joy to others’ adversities instead of feeling sympathy is considered socially undesirable or even malicious. In this vein, some researchers have used the terms “malicious joy” or “malicious pleasure” to describe schadenfreude (e.g., Leach et al., 2015; Cecconi et al., 2020). Others label schadenfreude as a “counter-empathic” reaction (e.g., Cikara et al., 2014; Hudson et al., 2019).

The opposite reaction to others’ misfortunes is sympathy, feeling concern or sorrow about distressful events in a person’s life (Clark, 2010). Thus, sympathy is an empathic reaction to the misfortune of others. In general, empathy refers to the processes in which the empathizer understands, feels, and shares another person’s world with self–other differentiation (Håkansson Eklund and Summer Meranius, 2021), and we consider sympathy an outcome of empathic processes. As schadenfreude and sympathy are both encountered in situations in which another person’s misfortune is observed, they are considered two sides of the same coin (Schindler et al., 2015).

To elucidate schadenfreude and sympathy in media reception, we will first dive into theories that focus on the elicitation of “counter-empathy” and schadenfreude specifically, before extending those insights to sympathy. In doing so, we bring together different research traditions that contribute to our understanding of schadenfreude. First, we lay the foundation with Affective Disposition Theory (ADT; e.g., Zillmann and Cantor, 1976, 1977; Zillmann, 2000, 2011, 2013; Raney, 2004), which is well established in media psychology. Second, we elucidate different approaches to investigating schadenfreude from a social psychology perspective (e.g., Wang et al., 2019). Interestingly, recent research on schadenfreude has neglected previous work in the line of ADT. For example, in an edited volume on schadenfreude (van Dijk and Ouwerkerk, 2014b), Zillmann, one of the founders of ADT, is mentioned in just two chapters (Ouwerkerk and van Dijk, 2014; van Dijk and Ouwerkerk, 2014c). This might be because Zillmann and Cantor did not initially use the term schadenfreude, although they described a similar emotion in the context of *counter-empathic reactions*. In later works, Zillmann (2000, 2011, 2013) included schadenfreude as a term. Therefore, we consider it even more important and beneficial to unite these different research traditions from media and social psychology perspectives. Additionally, we apply the appraisal perspective of emotion (e.g., Scherer et al., 2001) as a general framework. Finally, after examining individual appraisals and emotion elicitation, we consider the social dimension within emotion and emotion regulation. In conclusion, we propose a theoretical model that incorporates the factors involved in the elicitation and regulation of schadenfreude and sympathy in media reception. This article contributes to theory building in media psychology through the review of literature and theories to intellectually organize theoretical work (Slater and Gleason, 2012).

## 2 Theories about schadenfreude elicitation

In this section, we examine theories related to schadenfreude. We compare and unite ADT and different theories from social psychology that attempt to explain the occurrence of schadenfreude.

### 2.1 Affective Disposition Theory

The connection between ADT (Zillmann and Cantor, 1976, 1977; Raney, 2017) and schadenfreude was proposed by Zillmann (2000, 2011, 2013) in later works. We begin with a brief review of ADT and its development.

According to ADT (Zillmann and Cantor, 1976, 1977; Raney, 2017), entertainment experiences are heavily dependent on recipients’ attitudes toward media figures and the valence of events happening to them. Raney (2017) highlighted that the theory broadly consists of three psychological processes or components: 1) the dispositions formed and held toward a character, 2) the emotional reactivity to the plight of that character, and 3) the viewer’s hedonic response to the ultimate resolution of the narrative.

According to ADT, dispositions are built by moral judgments of the portrayed or implied behavior. Morally good and appropriate judgments lead to positive affective dispositions, whereas behavior appraised as morally wrong leads to negative affective dispositions. Positive affective dispositions lead to hope for a positive outcome for the character, whereas negative affective dispositions lead to contrasting anticipation and apprehension (hope for a negative outcome). In this process, viewers feel empathy or counter-empathy for the emotions experienced by a character. Importantly, this theory predicts that counter-empathy will increase when disliked characters experience negative outcomes. As a process theory, ADT considers that media usage processes often last longer (Vorderer et al., 2004). ADT has been widely applied and has received much empirical support (e.g., Grizzard et al., 2023a).

In the development of ADT, three perspectives elaborated in the next sections can be regarded as fundamental in understanding the generation of counter-empathy (*cf.* Raney, 2006). We want to highlight that these three perspectives follow the same underlying logic of ADT, as outlined above, but add value to certain contexts (humor, fiction, and sport; see also Zillmann, 2013).

#### 2.1.1 Disposition Theory of Humor

In the early 1970s, Zillmann and Cantor (1972) laid the foundation for disposition-based media theories. They studied why media users enjoy jokes that denigrate others. They used cartoons featuring typical status pairs relating to the household (parent–child), school (teacher–student), and office (employer–employee) where either the high- or low-status person was denigrated. The results showed that media recipients who were inferior to those who were denigrated perceived the joke as funny, while sharing a similar social status with people or groups who were denigrated led to no pleasure. Based on these results, the authors concluded that social standing leads to situationally formed affective dispositions toward people or groups. Specifically, being inferior to a person leads to a negative affective disposition toward that person. They further proposed that the intensity and valence of the affective disposition influence the empathic response toward the person. Furthermore, individuals tend to develop more empathetic responses toward people with whom they share relevant positive experiences, whereas the corresponding reaction is less evident with people who share relevant negative experiences. Therefore, the formation of affective dispositions depends on perceived experiential similarities. The implication of this theory is that pleasure (here, the joke) increases when the group to which the individual feels closest gains the upper hand (*cf.* Raney, 2006). Zillmann and Cantor (1972) therefore conclude by stating that “the *who disparages whom* is critical” (p. 198).

#### 2.1.2 Disposition Theory of Mirth or Drama

In the Disposition Theory of Mirth or Drama (1976), Zillmann and Cantor substantiated television viewers’ affective responses to a protagonist’s emotions. In the early 1970s, it was generally assumed that the emotional reactions of recipients toward media characters were based on empathic processes, whereby the viewer shares a feeling with the character (*cf.* Zillmann and Cantor, 1977). They revised this view by stating that “the observer’s emotional reaction may also be brought about by some aspect of the situation other than the witnessed emotional response *per se*, and that the observer’s response may or may not be similar to that of the performer” (Zillmann and Cantor, 1977, p. 156).

The Disposition Theory of Mirth or Drama can be seen as an extension of the originally formulated Disposition Theory of Humor because it is not aimed at humor only but at the (dis)similar experience of the emotions of others, whereby it does not matter “whether these others are protagonists in communications, public figures known only via the mass media, or friends and acquaintances encountered face-to-face in everyday situations” (Zillmann and Cantor, 1977, p. 164). Importantly, as a reason for (dis)similar experiences of emotion they considered affective dispositions toward media characters.

#### 2.1.3 Disposition Theory of Sports Spectatorship

On the basis laid out above, Zillmann et al. (1989) examined the affective reactions of recipients during sports broadcasts. In doing so, they explored people’s enjoyment of sports competitions. They stated that dispositions toward a character, athlete, or team are decisive for the entertainment experience of the recipient. They concluded that “enjoyment derived from witnessing the failure and defeat of a competing party increases with negative sentiments and decreases with positive sentiments toward that party” (p. 257).

#### 2.1.4 Extension of Disposition Theory

ADT was further expanded by Raney (2004), who added two propositions referring to the formation and maintenance of affective dispositions. First, “[t]he initial formation of an affective disposition toward a character may at times actually precede specific moral evaluations of the character” (p. 361). Story schemas of “good” and “bad” characters were proposed as guiding initial disposition formation. Second, viewers expect liked characters to do good things and disliked characters to do bad things. “[T]hose expectations lead viewers to interpret character actions and motivations in line with the established dispositional valences rather than to morally scrutinize each action and motivation” (Raney, 2004, p. 361).

In addition to ADT, Sanders (2010) argued that in forming character impressions, media recipients utilize both category- and attribute-based processing. Recipients begin by using categorical-based strategies, and only if the category assignment does not fit they will apply a reconciliation process using attribute-by-attribute analysis.

Taken together, throughout the historical development of ADT, various factors leading to affective dispositions have been identified, such as social status or experiential similarities, social norms, moral judgments, group affiliations, story schemas, and impression formation categories. However, most ADT studies describe moral judgment as the central process in affective disposition formation.

#### 2.1.5 Empathic and counter-empathic reactions

After disposition formation, it is assumed that positive affective dispositions lead to empathic reactions, whereas negative affective dispositions lead to counter-empathic reactions. Empathic reactions imply that the recipient feels *with* the protagonist and shares their emotions. Counter-empathic reactions indicate that the recipient does not show any empathic reaction and does *not feel with* the person. In contrast, the recipient feels deviating, even contrasting, emotions to the feelings of the person. Thus, experiencing pleasure at the negative outcome of a media figure (schadenfreude) is a form of counter-empathic reaction.

### 2.2 Types of schadenfreude

Schadenfreude appears in various mediated and non-mediated contexts. Wang et al. (2019) thoroughly reviewed schadenfreude in social psychology and identified three subtypes based on different needs or concerns (see Table 1). These three subtypes are distinguishable but interrelated:

1. Justice schadenfreude,
2. Aggression schadenfreude, and
3. Rivalry schadenfreude.^1^^,^^2^

**Table 1:** Schadenfreude types and formation of negative affective dispositions.

	Justice schadenfreude	Aggression schadenfreude	Rivalry schadenfreude
Main concern	Moral concern for social justice and fairness	Concern for social identity	Concern for social comparison and self-evaluation
Related theories	Deservingness theory	Intergroup theories	Social comparison theories
Formation of negative affective dispositions based on…	Moral evaluation, e.g., of norm violations or moral wrongdoings	Social identity, e.g., group affiliation, dislike of out-group, experiential similarities	Social comparison processes: upward comparison, e.g., malicious envy; downward comparison, e.g., need for positive self-evaluation, low self-esteem

The main concerns and related theories are based on those mentioned by Wang et al. (2019). Formation of negative affective dispositions based on… was added by the authors based on their review.

These three types of schadenfreude and their associated needs or concerns may inform the reasons for forming a negative affective disposition toward media characters that may go beyond classic moral considerations as proposed by the ADT. In the following sections, we describe the three subtypes and their associated psychological theories (see Table 1), compare them to ADT, and highlight their similarities.

#### 2.2.1 Justice schadenfreude, Deservingness Theory, and the Disposition Theory of Mirth

Justice schadenfreude occurs when a violation of a moral or social norm is restored by a “karmic” or justified misfortune. Here, Deservingness Theory plays a major role in explaining schadenfreude. Developed 20 years after the initial groundbreaking work by Zillmann and colleagues, Feather’s Deservingness Theory (Feather, 1994, 2014) states that deservingness is “a judgment which relates to outcomes that are earned because of a person’s actions or qualities” (Feather, 2006, p. 39). Feather (1994) described the conditions for a deserved or undeserved outcome. According to his analysis, two conditions are necessary for the deservingness of a negative outcome: a) responsibility for the action and b) a negatively valued action preceding the negative outcome. Feather elaborates on these two conditions and explains how moral judgments are closely related to deserving judgments. Taken together, Deservingness Theory predicts that when a person is responsible for a morally negative action, the negative outcome is perceived as deserved. For example, a student who cheats on an exam (a morally negative action for which he or she is responsible) deserves a failing grade (a negative outcome).

Deservingness Theory was later extended (Feather, 2006; Feather and McKee, 2009) to take discrete emotions into account. According to Feather and McKee (2009), schadenfreude results when a negative action by another person is followed by a negative outcome for that person because that outcome is perceived as deserved. In fact, the perceived deservingness of a misfortune has been identified as a key factor contributing to the experience of schadenfreude in many studies (e.g., van Dijk et al., 2009; Feather, 2014). It was even called the potential “royal road to schadenfreude” (Smith et al., 2009, p. 537). In general, the more a person is perceived as having deserved the misfortune, the more schadenfreude is felt.

This deservingness perspective is compatible with ADT, as Zillmann (2013) pointed out: “In accordance with dispositional considerations, judgments of deservedness yield hedonically divergent affective reactions to differently liked others’ success and failure. In particular, […] witnessing the failure of disliked persons who are judged deserving of the outcome prompted euphoric responses instead” (p. 138). Despite Zillmann’s acknowledgement of Feather’s Deservingness Theory, the notion of deservingness has not been systematically considered in ADT research (exceptions are Raney, 2002, 2005; Grizzard et al., 2023b).

Both the ADT and Deservingness Theory highlight the importance of moral evaluation. However, Feather and McKee (2009) noted that the values that build the basis for evaluations of an action “are not restricted to so-called moral values that relate to considering and caring for the welfare and interests of others and for society in general. They can span different value types, such as the range of value types proposed by (Schwartz (1992, p. 959)”. Additionally, they suggest like/dislike and in-group/out-group relations, as well as positive or negative self-evaluations, as potential moderators for judgments of deservingness, and thus already make a link to elements of other schadenfreude subtypes.

#### 2.2.2 Aggression schadenfreude, intergroup theories, and the disposition theories of humor and sports spectatorship

Social identity, often associated with an “us vs. them” distinction (e.g., Tajfel, 1982), is at the core of intergroup theories that inform schadenfreude research in the domain of aggression schadenfreude. Pleasure at the misfortune of an out-group member may serve as a sign to both in-group and out-group members that their interests are not aligned with those of the out-group (Cikara et al., 2011).

As Leach and Spears (2009) explained, “the classic view of schadenfreude […] suggests that it is the dysphoria at being outshone by a second party of similar status that is the most potent explanation of schadenfreude at this party’s subsequent misfortune” (p. 663). They found evidence for this assumption in a study with rival football teams during the 2000 European Championship. Simultaneously, they found evidence of schadenfreude regarding a third party’s failure that was not in direct competition with the in-group when the in-group had been defeated beforehand. Thus, negative feelings of being outshone by another group (the “pain of dejection,” p. 661) can lead to schadenfreude toward that or even a third group. Additionally, dislike of another sports team (out-group) was found to cause schadenfreude at its loss (e.g., Hareli and Weiner, 2002). Combs et al. (2009) investigated intergroup schadenfreude in politics and found that party affiliation and the extent to which participants identified with their parties predicted the strength of their schadenfreude.

This intergroup perspective on schadenfreude is compatible with the Disposition Theory of Sports Spectatorship (Zillmann et al., 1989). In addition, the role of groups was discussed in the first Disposition Theory of Humor (Zillmann and Cantor, 1972) with a focus on the disparagement of certain groups.

A relevant concept in the context of disparagement of out-group members is dehumanization. Dehumanization means denying or overlooking the humanity of others (Kteily and Landry, 2022). Over (2021) notes that out-group members may be denied some human qualities and states and that such dehumanization is thought to be associated with aggression toward out-group members which can be considered an extreme form of a counter-empathic reaction. In their tripartite model, Wang et al. (2019) propose dehumanization as the central mechanism for the occurrence of schadenfreude where the perceiver tends to dehumanize the victim by “temporarily losing the motivation to detect the victim’s mind, much like a psychopath” (p. 7). While they show that dehumanization and schadenfreude share similar predictors, direct empirical support for the notion of dehumanization within schadenfreude is still lacking.

#### 2.2.3 Rivalry schadenfreude, social comparison theory, and affective dispositions

Social comparison processes (Festinger, 1954) are thought to guide rivalry schadenfreude. It is generally assumed that another person’s misfortune provides a social comparison benefit relative to an earlier comparison between oneself and the person experiencing it (van Dijk and Ouwerkerk, 2014c). We divide this approach into two cases: upward comparison (envy) and downward comparison.

In the case of upward comparison (envy) and schadenfreude, it is important to distinguish between two forms of envy: malicious and benign. While both forms arise in situations where another person is better off and is accompanied by frustration, malicious envy includes the motivation to pull the other person down, but benign envy includes the motivation to move oneself up by improving one’s own performance (van de Ven et al., 2009). This distinction led to the prediction that malicious envy leads to schadenfreude, while benign envy does not, which found empirical support (van de Ven et al., 2015; Lange et al., 2018). Thus, schadenfreude occurs in situations where an upward social comparison leads to malicious envy that is resolved by a misfortune that “pulls down” the envied person.

In contrast, the theory of downward comparison (Wills, 1981) posits that people can enhance their self-evaluation by comparing themselves to a less-fortunate other. In the case of schadenfreude, a form of downward comparison occurs when one witnesses another person’s misfortune. Downward comparison theory further posits that downward comparison processes can be evoked by a threat to one’s psychological well-being. This may be the case for individuals with chronically negative self-evaluations or acute self-evaluation threats. Support for this notion was found by van Dijk et al. (2011a,b, 2012) who showed that schadenfreude was higher in individuals with low self-esteem and those who experienced a self-evaluation threat. Interestingly, this mechanism of downward social comparison is often considered in research on reality TV and schadenfreude (e.g., Hall, 2006; Hammes, 2016). van Dijk et al. (2012) used excerpts from the Dutch version of the music TV show American Idol in their study.

Integrating ADT, this could mean that negative affective dispositions are formed based on malicious envy (upward comparison) or the need for positive self-evaluation (social downward comparison). In the case of downward comparison, Wills (1981) mentioned similarity to the Superiority Theory of Humor (Zillmann and Cantor, 1976). He noted that humor stimuli commonly involve something about which the audience feels insecure, and that humor gives the audience “an opportunity to assuage their own insecurities through favorable comparison with another person’s misfortune, frustration, foolishness, imperfection, blundering, embarrassment, posturing, or stupidity” (p. 263).

In summary, Table 1 displays the different schadenfreude types and their related psychological theories and shows which factors can be derived for the formation of negative affective dispositions. Extending these insights to sympathy, we argue that the corresponding factors contribute to the formation of positive affective dispositions. For example, perceived individual similarity, in-group membership, and moral behavior should lead to liking a person, and thus feeling sympathy in the case of a misfortune.

## 3 Schadenfreude and sympathy in relation to other audience reactions

Schadenfreude and sympathy are other-focused emotions (Feather and McKee, 2009), another person (the media figure) is the focus of the emotion. As such, schadenfreude and sympathy are incompatible with identification which is “an imaginative process through which an audience member assumes the identity, goals, and perspective of a character” (Cohen, 2018, p. 261). To feel schadenfreude and sympathy toward a media figure, the audience needs to consider the media figure as a distinct person one can observe and judge which is not the case in identification.

Parasocial interactions and relationships, on the other hand, are compatible with the notion of schadenfreude and sympathy. In fact, schadenfreude has been included in a measure of parasocial interactions by Schramm and Hartmann (2008), capturing potential interpersonal processes between the audience and the media figure that take place during media exposure. A parasocial relationship, a cross-situational relationship a viewer or user holds with a media figure (Schramm and Hartmann, 2008), can be interpreted as a rather stable affective disposition (liking, dislike) toward a media figure, and thus might foster schadenfreude and sympathy accordingly. Similarly, homophily might influence affective disposition formation. Homophily is seen as “a subjective perception of similarity between oneself and another” (Tukachinsky and Tokunaga, 2013, p. 289) or as “the principle that a contact between similar people occurs at a higher rate than among dissimilar people” (McPherson et al., 2001, p. 416). In the context of affective dispositions, this means that a media figure that is perceived as similar would rather be liked than a media figure that is perceived as dissimilar. This is where the appraisal perspective becomes highly relevant, as will now be elucidated.

## 4 Schadenfreude and sympathy as individual audience emotions: an appraisal perspective

Taken together, several theories explain the emotions of schadenfreude and sympathy and identify the relevant factors of their elicitation within the individual. Appraisal theories are considered state-of-the-art approaches to studying emotions. They state that emotions are evoked by cognitive evaluations (appraisals) of events (e.g., Scherer et al., 2001) and that specific emotions result from a specific pattern of appraisals. Most approaches differentiate between primary and secondary appraisals. In primary appraisals, an event is typically assessed as a) significant or insignificant for further attention and b) relevant or irrelevant to personal goals. In the case of significance and relevance, secondary appraisals come into play, such as goal significance or coping potential (see, e.g., stimulus evaluation checks; Scherer et al., 2001).

Regarding emotions in media reception, Unz (2010) applied an appraisal framework to TV news. She noted that although media-induced emotions are processed in the same way as naturally occurring ones, the former may differ in some respects from the latter (Scherer, 1998). Presentation modes and editing (e.g., camera angles, cuts, and movements) may initiate and influence appraisal processes (e.g., novelty, intrinsic pleasantness, causality, and coping). For example, Grizzard et al. (2017) found that displaying high levels of graphic violence in a news story, as compared to low levels of graphic violence, elicited more intense moral emotions, such as contempt, anger, and moral disgust. In a similar vein, Lankhuizen et al. (2022) found that certain features of films (e.g., a reduction in perceived distance through close-ups and face depiction) can heighten the levels of empathy experienced by viewers. There is a substantial body of research on the influence of formal features of media, such as color, motion, or camera angle, on emotional responses (Detenber et al., 2021), which often focused on variables such as attention, arousal, or valence. However, research concerned with the influence of media features on appraisals is scarce.

In this section, we identify relevant appraisals with regard to schadenfreude.^3^ The event that is appraised and potentially leads to schadenfreude is the misfortune, harm, or damage to a person displayed in the media. First, to evoke schadenfreude, an event must be appraised as significant to attain further attention. We propose that audiences who pay attention to media content would comply with this appraisal. Second, the event must be appraised as relevant. We suggest that different concerns come into play here (moral concerns, concern for social identity, concern for social comparison, and self-evaluation; see Table 1). Thus, a misfortune can be appraised as being relevant regarding many different facets or predispositions. Third, the misfortune needs to be appraised as negative *for the media person*, which is, by implication of negative affective dispositions, considered positive *for the observer*. For secondary appraisals, we propose deservingness as the central appraisal, as deservingness has been shown to be the key to schadenfreude in many previous studies (e.g., Smith et al., 2009; Van Dijk et al., 2009; Feather, 2014). Also, Zillmann (2011) proposed that the delight when witnessing the victimization of a disliked antagonist increases with “the extent to which the antagonist is deemed deserving of a particular victimization” (p. 110). We further propose that deservingness appraisals are influenced by the severity of the misfortune. Schadenfreude has been empirically linked to minor harm rather than to severe harm (e.g., Hareli and Weiner, 2002; Schumpe and Lafrenière, 2016). Furthermore, considering the context of justice schadenfreude, an equitable, and thus deserved, retribution (e.g., Grizzard et al., 2021) means that the severity of the misfortune should be concordant with the severity of the moral transgression. In addition, Leach et al. (2015) compared appraisals of schadenfreude and gloating and concluded that “schadenfreude was characterized by appraisals that others, rather than the self, were the agent of the precipitating event. Schadenfreude was also unique in being experienced as a state of lower power and performance” (p. 7).

Hess (2018) argued that for feeling schadenfreude an additional step of appraisal is necessary, namely “an appraisal of the presumed appraisal of the event by the protagonist” (p. 307) who suffers from the misfortune. “That is, observers first have to appraise an event from the perspective of a person who, for example, slips on a banana peel, and then they appraise the outcome with regard to their own goals” (p. 307). This step is one way to consider the social situation in which schadenfreude is usually embedded, and it relates to the concept of social appraisal (Manstead and Fischer, 2001).

For appraisals that lead to sympathy, the misfortune must be appraised as significant and relevant too. The event also needs to be appraised as negative *for the media person*. Contrary to schadenfreude and negative affective disposition, the misfortune here is not considered positive *for the observer* but rather negative. For secondary appraisals, we propose deservingness as the central appraisal. Studies have shown that sympathy is more likely when a misfortune is appraised as not deserved (e.g., Feather et al., 2013; Tscharaktschiew and Rudolph, 2016). With regard to the severity of misfortune, studies show that sympathy is more likely when the amount of harm is severe (e.g., Schindler et al., 2015).

## 5 Social dimensions within schadenfreude and sympathy

While this article has so far mainly focused on schadenfreude and sympathy based on individual appraisal, we should not neglect that media recipients are often not in solitude, but rather in company and engaging in communicative action with other people. Thus, it is vital to consider the social situation in which emotions arise.

### 5.1 Schadenfreude as a social-functional dominance regulator

Lange and Boecker (2018) elaborated on the interpersonal and social functions of schadenfreude. They argued that schadenfreude arises in the context of power and dominance, and that it contributes to the regulation of hierarchical differences between the self and others, specifically to the reduction of a superior other’s dominance. Importantly, this does not apply to simple status differences that may be respected by inferiors but to dominance hierarchies that are rather fixed. In their empirical investigations, Lange and Boecker found evidence that schadenfreude is a response to a misfortune happening to an initially dominance-displaying person, and that the public expression of schadenfreude leads to a reduction in the other person’s dominance, confirming their argument. This argument corresponds to Zillmann and Cantor’s (1972) original Disposition Theory of Humor. Lange and Boecker (2018) further indicated that, in other non-hierarchical contexts, the expression of schadenfreude might not be approved or may even backfire. They stated that “the legitimacy of the expression of schadenfreude might be a boundary condition of its social function” (p. 12).

This is vital because individuals are fundamentally engaged in social constellations. Therefore, we argue that, when studying audience emotions, one must consider the social dimension of appraisals (Manstead and Fischer, 2001; Döveling, 2012; Döveling and Sommer, 2012).

### 5.2 Social dimension within appraisals

Previous research (Döveling, 2012) suggests that social appraisal must be considered when addressing schadenfreude and sympathy. For example, Rimé et al. (1992) found that most emotional experiences were shared with others shortly after their occurrence. The authors noted that *social sharing* is a fundamental part of the emotional experience (see also Christophe and Rimé, 1997; Rimé et al., 1998; Pennebaker et al., 2001). Therefore, the social sharing of emotional judgments is an integral component of emotional experiences and directly influences one’s own appraisal and reappraisal of not only media-related information but also one’s own emotional assessment of mass-mediated messages.

Additionally, *social appraisal* processes must be considered. Manstead and Fischer (2001) pointed out that it is crucial “that individuals anticipate others’ definitions of the situation and how they are likely to respond to the situation and to our behaviors” (p. 224) in order to coordinate social activity and maintain social bonds. Hence, any social event—and media use needs to be considered a social event as well—is evaluated by others. In this sense, “it is not only an event that is evaluated in relation to the self; it is also very likely to be appraised in relation to the reactions of others” (Manstead and Fischer, 2001, p. 224). In this context, social norms are vital because they can *hinder* or *foster* the authentic display of emotion, especially in social situations with strong social norms. Through mixed-method research involving observational data, Döveling (2012) revealed the effects of co-viewing on the emotion regulation and management processes of schadenfreude and sympathy. In this process, participants’ emotional reactions and those of relevant others were socially shared and appraised. Discussing appraisals with others, especially with relevant others such as peer groups and friends, can set rule reminders (Hochschild, 1979) in social situations. As a result, a process of reappraisal of participants’ emotions was observed that tended to lead to greater sympathy and less schadenfreude.

Here, we postulate that interpersonal negotiation processes are engendered, which needs to be understood as an important social factor, as they help recipients affiliate with their peer group by adjusting and adapting their own feelings within social interactions. Döveling and Sommer (2012) summarized these processes under the term *socio-emotional meta-appraisal* (SEMA) in media reception. Whereas Bartsch et al. (2008) mentioned meta-emotion and emotion regulation from an appraisal perspective, Döveling and Sommer (2012) highlighted the social aspects in emotion regulation.

## 6 The Model of Individual and Social Appraisals of Misfortunes of others

Based on the presented conceptual analysis, we developed an integrative model, which identifies core factors involved in the elicitation and regulation of schadenfreude and sympathy in media reception. Before presenting the process model in detail, we first describe the cornerstones of its development whereby we pursued the goal of integrating different theoretical perspectives. First of all, the logic of ADT was considered to be fundamental. That is, in the case of a negative event happening to a media figure, negative and positive affective dispositions (dislike and liking) lead to schadenfreude and sympathy, respectively. Next, the different schadenfreude types were used as relevant information about how affective dispositions are built. Importantly, we considered affective dispositions as the result of the appraisal of the media figure who (later) experiences a misfortune. In particular, the analysis outlined above identified moral concerns (justice schadenfreude), group concerns (aggression schadenfreude), and individual concerns (rivalry schadenfreude) that guide the appraisal of the media figure experiencing a misfortune. Additionally, in the appraisal of the media figure certain media features could also exert an influence. Subsequently, for the appraisal of the misfortune and in line with schadenfreude research, deservingness was highlighted as a key appraisal, and the appraisal of the severity of misfortune was further added. Up until this point, factors refer to *individual appraisals*, as media recipients perceive, and judge media content based on their primary and secondary appraisals. In the next step, factors were added, that revolve around the social situation wherein emotions arise and which might lead to re-appraisal and a change of emotion. The model takes into account components as proposed by SEMA. In particular, these are norms as well as the anticipation of others’ judgment of one’s own appraisal and corresponding emotion (*social appraisal*) and the exposed and openly communicated appraisals and corresponding emotions by others (*social sharing*). Finally, similar feedback loops as in ADT were incorporated (see Zillmann, 2013), indicating that the process is recursive and can be repeated several times and that previous experiences might influence future encounters with a media figure.

**Figure 1:**
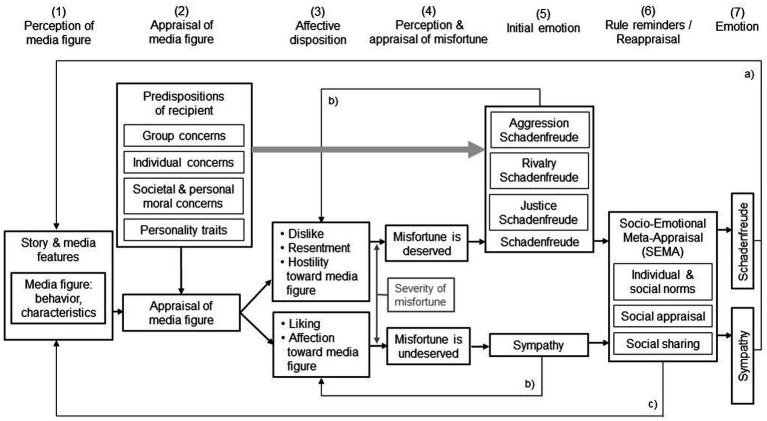
The Model of Individual and Social Appraisals of Misfortunes of others (MISAM) shows factors involved in the elicitation and regulation of schadenfreude and sympathy in media reception.

As a result, we propose the *Model of Individual and Social Appraisals of Misfortunes of others* (MISAM; see Figure 1), which identifies factors involved in the elicitation and regulation of schadenfreude and sympathy in media reception. The focus of the model is on the recipient and the psychological processes that take place within the individual. The main process and key factors identified here apply across different media (e.g., traditional media, social media). A media figure is any figure in the media, and we consider the terms media figure, media character, media person, or media persona synonymous. A media figure might be a real or fictional character and the term also applies to human-like entities such as animated animals, as long as the media figure can be evaluated based on their characteristics or actions. Inanimate objects do not fulfill this criterion. The process model is described in detail in the following sections.

The MISAM was designed as a process model. In the first part of the model, the media figure or media figure’s behavior is perceived (1) and appraised (2), and this appraisal process results in a negative or positive affective disposition (3). In the following section, we propose specific relationships between the variables in this part of the model.

If the media figure is portrayed as a member of a certain group and the recipient holds a certain group identity (e.g., a political party), the out-group membership of the media figure (e.g., an opposing political party) leads to dislike and hostility, whereas in-group membership (e.g., the same political party) leads to liking and affection. If the recipient has individual concerns, such as low self-esteem (van Dijk et al., 2011b) or feels envious toward the media figure [e.g., as the person is portrayed as successful, rich, or of high status, such as in the tall poppy research by Feather (1994)] the recipient will show dislike or hostility toward the media figure. If the media figure violates social or personal norms (e.g., harming another person or lying), it also results in dislike and hostility. Personality traits also influence the appraisal process. For example, recipients with a high social dominance orientation are more likely to feel dislike and hostility toward low-social-status groups (Hudson and Uenal, 2023), and recipients high in agreeableness are more likely to feel positive toward other people (e.g., Greenier, 2018). These appraisals refer to media figures embedded in a story, as well as in a media format with corresponding features that might influence the appraisal process. Certain narratives activate story schemas that guide disposition formation: “heroes” are liked and “villains” are disliked. As media features (e.g., camera movement) have not been well studied with regard to appraisal processes and disposition formation, we will refrain from making concrete predictions at this point.

In the next part of the process model, a misfortune occurs and is appraised regarding its deservingness (4), resulting in schadenfreude or sympathy (5). Specifically, the appraisal of deservingness is highly influenced by the valence of the affective disposition toward the media figure. If the media figure is disliked (negative affective disposition), the misfortune is perceived as deserved. If the media figure is liked (positive affective disposition), the misfortune is perceived as not deserved. Another factor influencing deservingness appraisal is the severity of the misfortune. Generally, severe harm is considered to be less deserved than mild harm. Additionally, we assume for the path of dislike that the intensity of dislike and the amount of harm interact and that a high intensity of dislike would allow for severe harm to still be considered deserved and acceptable (e.g., a murderer getting seriously injured); this is known as equitable retribution (e.g., Grizzard et al., 2021). Deservingness appraisal, then, is closely linked to the resulting emotion: a deserved misfortune evokes schadenfreude, and an undeserved misfortune leads to sympathy toward the media figure. It is assumed that the intensities of deservingness appraisals and schadenfreude are positively correlated. A negative correlation is assumed between deservingness and sympathy. Depending on which predisposition has the main influence in the appraisal of the media figure (Step 2), different types of schadenfreude are distinguished (grey arrow). Group concerns constitute aggression schadenfreude, individual concerns constitute rivalry schadenfreude and moral concerns constitute justice schadenfreude.

In the subsequent part of the model, this emotion is potentially reappraised (6), and the final emotion results (7). In social situations, the resulting emotion is reappraised in light of individual or social norms regarding the emotions of schadenfreude and sympathy and their display (e.g., social desirability), in light of anticipated emotions by others (social appraisals) and of the social sharing of emotions by others. Schadenfreude is displayed when social factors foster its display (e.g., other individuals cheering at the misfortune) or when no social factors hinder its display (e.g., no negative comments from others about how they disapprove of schadenfreude). If these factors or rule reminders work in opposite directions (e.g., other individuals voicing sympathy), sympathy may occur. This results in the final emotion (7).

Feedback loop a) indicates that the process is recursive and can be repeated several times. Feedback loop b) indicates that the feeling of schadenfreude increases dislike (negative affective disposition) toward the media figure or their social group in future encounters, because this experience then serves as a kind of previous attitude (predisposition) held by the recipient. This holds equally true for feelings of sympathy and liking in future encounters. Feedback loop c) indicates that socio-emotional meta-appraisal influences future media reception situations and appraisal processes within them (e.g., an emotion shared within a group contributes to social norm perceptions in the future).

## 7 Conclusion and implications for future research

In this article, we reviewed and integrated ADT and recent work from social psychology and used an appraisal framework to conceptualize the elicitation and regulation of schadenfreude and sympathy in media reception. Our thoughts focused on misfortunate events happening to figures in the media and on schadenfreude and sympathy as the resulting emotions in the recipient. As a result of integrating relevant theoretical work from different disciplines, we proposed the Model of Individual and Social Appraisals of Misfortunes of others (MISAM). The MISAM organizes important factors in the elicitation and regulation of schadenfreude and sympathy in media reception. Thus, this article contributes to theory building in media psychology through the review of literature and theories and attained the goal of intellectually organizing theoretical work (Slater and Gleason, 2012).

Coming from a media psychological perspective, ADT (Zillmann, 2013) is a central theory that we built upon. However, integrating psychological and sociological findings complements ADT in at least three relevant ways and improves the understanding of schadenfreude and sympathy in media reception.

First, by considering the three types of schadenfreude (Wang et al., 2019) and their associated needs or concerns, we were able to identify reasons for forming a negative affective disposition toward characters that go beyond the classic moral considerations propagated by ADT. Although other factors have been mentioned throughout the historical development and extension of ADT, it has most often been concerned with moral evaluation. Besides moral concerns, the MISAM includes individual and group concerns and thus extends the view on which factors are relevant for the formation of affective dispositions.

Second, we added the appraisal of deservingness of the misfortune as a central factor leading to schadenfreude and sympathy. While Zillmann (2013) mentioned judgments of deservingness in his text, he never incorporated them into his visualizations of ADT. In the MISAM, deservingness is highlighted as a key factor for schadenfreude and sympathy. The MISAM also deviates from the ADT model by Zillmann (2013) in some respects. In particular, we omitted the anticipatory emotion step. While anticipatory emotions are relevant to feelings of suspense and entertainment, they are not necessary to feel schadenfreude or sympathy, the primary outcomes of our model.

Third, by adopting an appraisal perspective, the MISAM ties in with state-of-the-art emotion research. Importantly, the model not only considers individual appraisals but also incorporates the social situation in which emotions arise. Even when no other person is present, but especially when consuming media content with others, the anticipation of how others might react to one’s own emotional display can influence affective reactions to a stimulus. This seems especially true in schadenfreude, as this emotion is sometimes considered socially undesirable. Therefore, it is important to consider social factors, such as norms, social appraisal, and social sharing. By doing so, the MISAM applies a holistic view of emotion elicitation and regulation in media reception and highlights the importance of social factors.

Whereas we highlight deservingness as a key appraisal in the process of schadenfreude elicitation, Wang et al. (2019) propose dehumanization as the primary mechanism. We believe that dehumanization might play a role in certain schadenfreude contexts (e.g., intergroup context, hate speech), but bears less relevance in other schadenfreude contexts (e.g., individual context, social comparison). Additionally, Kteily and Landry (2022) point out that dehumanization and dislike sometimes are muddled and mention the “potential for any effects of dehumanization to be confounded with those of dislike” (p. 234). Thus, further research in this regard and in relation to schadenfreude is necessary and, therefore, we did not include dehumanization in the MISAM.

Some parts of the MISAM are based on established theories, while others are preliminary. First evidence for core mechanisms of the model was presented by Döveling and Suter (2023). Open questions remain regarding the impact of media features on disposition formation, appraisal processes, and the resulting emotions. Future research could identify specific modes or techniques (e.g., Unz, 2010) that have an influence here. Future research could additionally investigate if the experiences of absorption or transportation during media reception intensify schadenfreude and sympathy responses. Although the emotion of schadenfreude is differentiated into several subtypes, sympathy is still conceptualized quite straightforwardly. Scholars could consider whether a similar differentiation of subtypes is possible in the emotion of sympathy. The appraisal of deservingness is thought to be highly dependent on the affective disposition toward a media figure experiencing a misfortune. Nevertheless, other aspects of the misfortunate situation (e.g., severe harm or damage) might influence this appraisal process, in some cases leading to outcomes other than schadenfreude despite a negative affective disposition.

Overall, the MISAM opens arrays for further research in media psychology, such as parasocial interaction and emotion research. Furthermore, we conclude that the proposed model holds value beyond the entertainment narratives context. Scholars have recently begun to investigate communication involving schadenfreude on social media (e.g., Cecconi et al., 2020; Grizzard et al., 2023b) or political communication (e.g., Nai and Otto, 2020). Future research could benefit from incorporating the MISAM as it displays relevant variables in the elicitation and regulation of schadenfreude and sympathy (e.g., predispositions, schadenfreude types, and reappraisal in a social context), leading to a deeper understanding of emotions in media reception.

## Footnotes

^1^Three similar explanations were already outlined by Smith et al. (2009): “schadenfreude resulting from personal gain” (e.g., in sports and politics) can be linked to aggression schadenfreude, “schadenfreude resulting from deserved misfortunes” can be linked to justice schadenfreude, and “schadenfreude resulting from envy” can be linked to rivalry schadenfreude.

^2^Another typology was proposed by Cecconi et al. (2020). “Compensation” can be linked to rivalry schadenfreude, “identification” can be linked to aggression schadenfreude, and “injustice schadenfreude” can be linked to justice schadenfreude. They proposed to subsume compensation (rivalry) and identification (aggression) under the term “image schadenfreude.” They further proposed a fourth, distinct type: “aversion schadenfreude,” where the notion of dislike “simply due to the way he is or behaves” (p. 6) is central to feeling pleasure at the misfortune of others.

^3^Although Zillmann (2011) titled one chapter “An appraisal paradigm for media characters and the emotions they evoke” (p. 109), he did not situate his analysis within appraisal theory, e.g., he did not specify primary or secondary appraisals.
